# Gremlin in the Vitreous of Patients with Proliferative Diabetic Retinopathy and the Downregulation of Gremlin in Retinal Pigment Epithelial Cells

**DOI:** 10.1155/2020/9238742

**Published:** 2020-04-12

**Authors:** Dong Qin, Yan-rong Jiang, Zijun Meng

**Affiliations:** ^1^Henan Eye Institute, Henan Provincial Eye Hospital, People's Hospital of Zhengzhou University, Zhengzhou, China; ^2^Department of Ophthalmology, People's Hospital, Peking University, Beijing, China

## Abstract

Diabetic retinopathy (DR) is one of the most common causes of blindness globally. Proliferative DR (PDR), an advanced stage of DR, is characterized by the formation of fibrotic membranes at the vitreoretinal interface. The proliferation, migration, and secretion of extracellular matrix molecules in retinal pigment epithelial (RPE) cells contribute to the formation of fibrotic membranes in PDR. Gremlin has been reported to be upregulated in response to elevated glucose levels in the retina of diabetic rat and bovine pericytes. However, the role of gremlin in PDR remains unclear. In the present study, the vitreous concentrations of gremlin were significantly higher in the PDR (67.79 ± 33.96) group than in the control (45.31 ± 12.31) group, and high glucose levels induced the expression of gremlin in RPE cells. The elevated expression of extracellular matrix molecules, such as fibronectin and collagen IV, was significantly reduced by gremlin siRNA in human RPE cells under high-glucose conditions. Thus, gremlin may play a vital role in the development of PDR.

## 1. Introduction

Diabetic retinopathy (DR) is the major cause of adult blindness globally [1]. Sustained high glucose levels play a vital role in the development of DR. Proliferative DR (PDR), an advanced stage of DR, is characterized by the formation of fibrotic membranes at the vitreoretinal interface. Retinal pigment epithelial (RPE) cells, which lie between Bruch's membrane and the retina, create the outer blood-retinal barrier (OBRB) and play a vital role in the pathological processes that lead to the vision loss. Extracellular matrix molecules, in combination with RPE cells and other cell types, are the major components of fibrotic membranes [2–4].

Gremlin is a secretory protein composed of highly conserved 184 amino acids [5–8]. This protein is a member of the structural cysteine knot superfamily and is present in both soluble and cell-associated forms [9–12]. Gremlin belongs to a new family of bone morphogenetic protein (BMP) antagonists that is involved in a number of physiological processes, such as growth, differentiation, survival, and development of cells [5, 9–13]. It has been reported that gremlin contributes to a number of diabetic fibrotic diseases [5, 9–14]. Gremlin has also been reported to be localised to the retina of diabetic rats, and the high levels of its expression have been demonstrated in bovine retinal pericytes under high-glucose conditions, compared with the control group [15].

Despite the demonstrated association between gremlin and diabetic diseases, information about the potential role of gremlin in PDR remains limited. The present study illustrated that the vitreous concentrations of gremlin were significantly elevated in the PDR group compared to the control group. The expression of fibronectin, collagen IV, and gremlin was upregulated in RPE cells under high-glucose conditions. In addition, the high expression of fibronectin and collagen IV was blocked by gremlin siRNA in RPE cells under high-glucose conditions.

## 2. Materials and Methods

### 2.1. Collection of Vitreous

We assayed gremlin levels in the vitreous from 48 eyes (48 individuals) with either proliferative vitreoretinopathy (PDR group, 26 eyes) or idiopathic epimacular membrane (control group, 22 eyes) all of whom had undergone pars plana vitrectomy. This part of the study was conducted in accordance with the Declaration of Helsinki. We received institutional approval from the review committee of Henan Provincial Eye Hospital. Informed consent was obtained from each subject for all examinations and procedures. At the beginning of the removal of the vitreous, approximately 0.8 mL of undiluted vitreous was aspirated through the vitreous cutter. The vitreous samples were centrifuged for 10 minutes (4°C, 3000 rpm) and immediately stored at -80°C. The concentrations of gremlin were determined using enzyme-linked immunosorbent assay (ELISA).

### 2.2. Reagents

Gremlin antibody was obtained from Abcam (Danvers, MA). Gremlin, fibronectin, and collagen IV ELISA kits were purchased from Elabscience Biotechnology Co., Ltd (Wuhan, China). *β*-Actin antibody, human gremlin siRNA, and siRNA control were obtained from Guangzhou RiboBio Co., Ltd (Guangzhou, China).

### 2.3. Cell Culture

Human RPE cell line (ARPE-19; CRL-2302) was obtained from the American Type Culture Collection (Manassas, VA, USA), and the cells were cultured in Dulbecco's modified Eagle medium (DMEM) (Gibco, Grand Island, NY, USA). The medium was supplemented with 10% foetal bovine serum (FBS), 100 ng/mL streptomycin, and 100 U/mL penicillin. The cells were maintained in a humidified incubator (37°C, 5% CO_2_).

### 2.4. Real-Time Polymerase Chain Reaction (PCR) Analysis

A TRIzol reagent kit was used to extract total RNAs from human RPE cells. The cDNA synthesis was performed using a RevertAid First Strand cDNA Synthesis Kit (Fermentas, St. Leon-Rot, Germany). Real-time PCR was determined with the ABI Sequence Detector System 7500 (Applied Biosystems, Carlsbad, CA). The primers were as follows: for human fibronectin, forward 5′-GAT AAA TCA ACA GTG GGA GC-3′ and reverse 5′-CCCAGA TCA TGG AGT CTT TA-3′; for human collagen IV, forward 5′-AGA GTC AGC ATC GGC TAC CT-3′ and reverse 5′-AGG AAG GGC ATG GTG CTG AA-3′; for human GAPDH, forward 5′-TGT TCG ACA GTC AGC CGC AT-3′ and reverse 5′-ACT CCG ACC TTC ACC TTC CC-3′; and for human gremlin, forward 5′-AAG CGA GAC TGG TGC AAA AC-3′ and reverse 5′-CTT GCA GAA GGA GCA GGA CT-3′. The reaction conditions are as follows: initial denaturation at 95°C for 10 min, followed by 39 cycles of 95°C for 15 s and 60°C for 30 s. The mRNA expression was calculated to the level of GAPDH mRNA.

### 2.5. Western Blot Analysis

Western blot analysis was performed as described previously [16, 17]. In brief, 20 *μ*g of protein was analysed by electrophoresis on 10% sodium dodecyl sulfate polyacrylamide electrophoresis (SDS-PAGE) gels and transferred to polyvinylidene fluoride (PVDF) membranes (Millipore, Billerica, MA). Specific bands were visualized using an enhanced chemiluminescence detection system (Amersham, Arlington Heights, IL). The primary antibodies of gremlin were used at a dilution of 1 : 250.

### 2.6. ELISA Analysis

After the samples were collected, the levels of gremlin in the vitreous were measured using a gremlin ELISA kit. In addition, the protein levels of fibronectin and collagen IV in the culture supernatants of the RPE cells were measured using fibronectin and collagen IV ELISA kits according to the manufacturer's instructions.

### 2.7. RNA Interference

The RPE cells were transfected with control siRNA or gremlin siRNA (50 nM each). The cells were transfected with riboFECT™ CP reagent, according to the manufacturer's protocol.

The cells were transfected with siRNA for 24 h and then incubated in the presence of high glucose for an additional 24 h. The gremlin siRNA sequence is CCA CCT ACC AAG AAG AAG A.

### 2.8. Statistical Analysis

Statistical analysis was completed using a one-way analysis of variance followed by Tukey's test, the Mann–Whitney *U* test, and the chi-squared test. SPSS 17.0 (SPSS, Chicago, IL, USA) was used for analysis. All the data are expressed as mean ± standard deviation (SD), and a *p* value < 0.05 was considered statistically significant.

## 3. Results

### 3.1. Vitreous Concentration of Gremlin in Individuals with PDR and Idiopathic Epimacular Membrane

**Figure 1:**
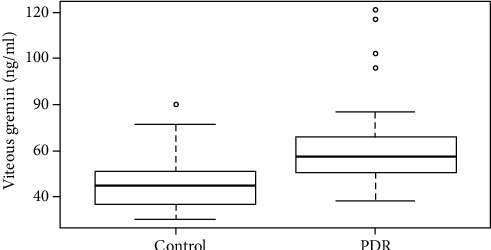
The vitreous concentration of gremlin in patients with PDR and idiopathic epimacular membrane. Box plots showed that the vitreous concentration of gremlin in patients with PDR was significantly higher than in patients with idiopathic epimacular membrane (*p* < 0.001). PDR represents proliferative diabetic retinopathy; control represents idiopathic epimacular membrane.

**Figure 2:**
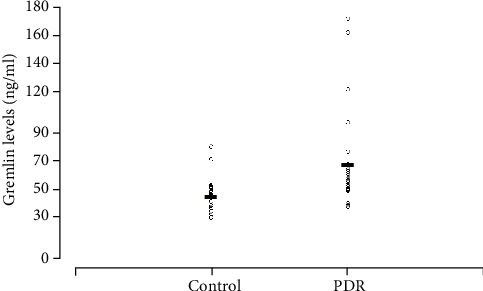
Scatter plot showed the levels of gremlin in the vitreous from the eyes with proliferative diabetic retinopathy (PDR, *n* = 26) and idiopathic epiretinal membrane (control samples, *n* = 22). Open circles represent the vitreous levels of gremlin. The horizontal lines indicate the mean concentration of gremlin in each group.

The study included 48 individuals, with 26 individuals in the PDR group and 22 individuals in the control group (Table 1). Individuals in the PDR group were significantly younger than the individuals in the control group, and there was no significance in the individuals' sex (Table 1). Fasting blood glucose levels in the PDR group were significantly higher than in the control group (Table 1). An ELISA kit was used to detect the concentration of gremlin in individuals with PDR and in those with idiopathic epimacular membrane. The vitreous concentration of gremlin was significantly higher in the PDR (67.79 ± 33.96) group than in the control (45.31 ± 12.31) group (Figures 1 and 2).

### 3.2. Induction of Fibronectin and Collagen IV under High-Glucose Conditions in RPE Cells

**Figure 3:**
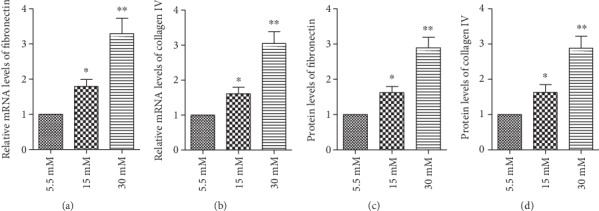
High glucose levels induced the expression of fibronectin and collagen IV in RPE cells. Before measuring the expression of fibronectin and collagen IV, the cells were exposed to normal glucose (5.5 mM) and high glucose (15 mM or 30 mM) for 24 h. A real-time PCR and an ELISA kit analysis illustrated that the (a, b) mRNA and (c, d) protein levels of fibronectin and collagen IV were upregulated in response to high glucose compared with normal glucose. The data shown represent the mean ± standard deviation (SD) of three independent experiments. ^∗^*p* < 0.05 versus 5.5 mM; ^∗∗^*p* < 0.01 versus 5.5 mM.

To examine the expression of fibronectin and collagen IV under high-glucose conditions, the cells were cultured in a medium containing normal glucose (5.5 mM) and high glucose (15 mM or 30 mM) and were exposed for 24 h. Real-time PCR and ELISA kit data revealed an elevated mRNA and protein level of fibronectin and collagen IV in the cells under high-glucose conditions (Figures 3(a)–3(d)).

### 3.3. High Glucose Induced the Expression of Gremlin in FPE Cells

**Figure 4:**
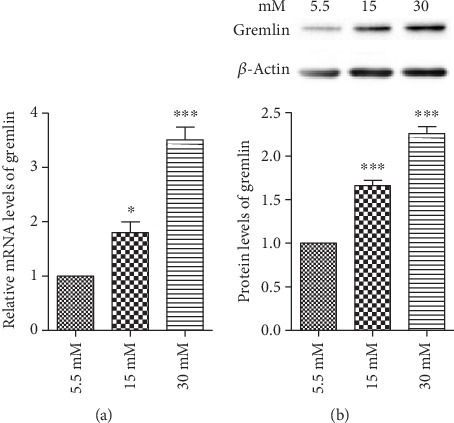
High glucose induced the expression of gremlin in RPE cells. The cells were exposed to normal glucose (5.5 mM) and high glucose (15 mM or 30 mM) for 24 h. A real-time PCR and a western blot analysis illustrated that the (a) mRNA and (b) protein levels of gremlin were upregulated in response to high glucose compared with normal glucose. The data shown represent the mean ± SD of three independent experiments. ^∗^*p* < 0.05 versus 5.5 mM; ^∗∗∗^*p* < 0.001 versus 5.5 mM.

**Figure 5:**
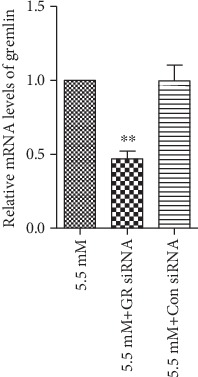
Gremlin siRNA significantly downregulated the expression of gremlin in RPE cells under normal glucose conditions. The cells were transfected with siRNA for 24 h and then incubated in normal glucose for an additional 24 h. A real-time PCR analysis showed that the silence of gremlin significantly reduced the expression of gremlin in RPE cells. The data represent the mean ± SD of three independent experiments. ^∗∗^*p* < 0.01 versus 5.5 mM. GR represents gremlin; Con represents control.

To examine the expression of gremlin under high-glucose conditions, the cells were cultured in a medium containing normal glucose (5.5 mM) and high glucose (15 mM or 30 mM) and were exposed for 24 h. Real-time PCR and western blot data revealed an elevated mRNA and protein level of gremlin in the cells under high-glucose conditions (Figures 4(a) and 4(b)). In addition, using real-time PCR, we found that gremlin siRNA significantly downregulated gremlin expression in RPE cells under normal glucose conditions (Figure 5).

### 3.4. Effect of Gremlin siRNA on High Glucose-Induced Fibronectin and Collagen IV in Human RPE Cells

**Figure 6:**
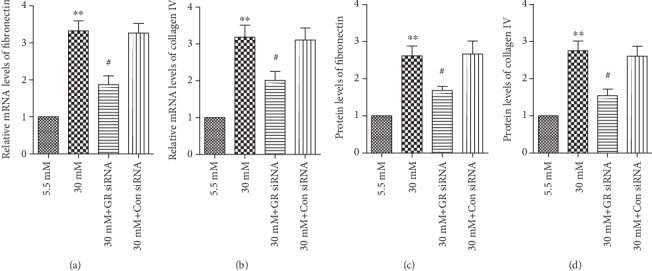
Downregulation of gremlin reduced the expression of fibronectin and collagen IV in RPE cells under high-glucose conditions. The cells were transfected with siRNA for 24 h and then incubated in the presence of high glucose for an additional 24 h. Real-time PCR and an ELISA kit analysis showed that the silence of gremlin significantly downregulated the expression of (a, c) fibronectin and (b, d) collagen IV in RPE cells under high-glucose conditions. The present data represent the mean ± SD of three independent experiments. ^∗∗^*p* < 0.01 versus 5.5 mM; ^#^*p* < 0.05 versus 30 mM. GR represents gremlin; Con represents control.

Having found elevated gremlin levels in the vitreous of patients with PDR and in RPE cells under high-glucose conditions, we examined the effect of gremlin siRNA on fibronectin and collagen IV in RPE cells under high-glucose conditions. The cells were transfected with siRNA for 24 h and then incubated in the presence of high glucose (30 mM) for an additional 24 h. Real-time PCR and an ELISA kit analysis showed that gremlin siRNA significantly reduced the mRNA and protein levels of fibronectin and collagen IV in RPE cells under high-glucose conditions (Figures 6(a)–6(d)).

## 4. Discussion

Prolonged hyperglycaemia is the major risk factor for the pathogenesis of DR. Prolonged hyperglycaemia activated cytokines, growth factors, and other molecules. The high levels of extracellular matrix molecules were detected in many organs of patients with diabetes mellitus and cell types under elevated glucose conditions [18–21]. It has been reported that extracellular matrix molecules contribute to the thickening of the basement membrane and the formation of fibrotic membranes during the development of DR [2]. Extracellular matrix molecules are upregulated in fibrotic membranes from PDR patients [22] and can be mediated by growth factors such as transforming growth factor beta (TGF-*β*) and endothelin 1 (ET-1) [23, 24]. In addition, our previous study also demonstrated that high glucose levels induced the expression of extracellular matrix molecules in RPE cells [25]. Increased extracellular matrix molecule deposition is a structural hallmark of DR [23, 26–29].

Gremlin has been implicated in the pathogenesis of human diseases, such as idiopathic pulmonary fibrosis, pulmonary hypertension, and diabetic nephropathy [30, 31]. In addition, gremlin is involved in human tumours [32, 33] and is expressed by tumour endothelium in vivo and by proangiogenic endothelial cells in vitro [34]. Gremlin stimulates endothelial cell intracellular signalling pathways and migration in vitro, leading to an angiogenic response in vivo [34, 35]. Gremlin is also proven to be localised to the outer retina of diabetic rats, and its high expression has been demonstrated in bovine retinal pericytes under elevated glucose conditions compared with the control group [15]. In the present study, the vitreous levels of gremlin were 67.79 ± 33.96 ng/mL in PDR and 45.31 ± 12.31 ng/mL in the control samples. Significant differences were found between the PDR and control groups. The vitreous gremlin levels in the eyes with PDR were significantly higher than the levels in the eyes with idiopathic epimacular membrane. In addition, high glucose induced the expression of fibronectin, collagen IV, and gremlin in a dose-dependent manner, and the high expression of fibronectin and collagen IV was significantly reduced by silence of gremlin in human RPE cells. The findings indicate increased vitreous levels of gremlin in the eyes with PDR, high levels of gremlin, fibronectin, and collagen IV in high glucose-treated RPE cells, and blockage of gremlin inhibition of fibronectin and collagen IV in RPE cells under high-glucose conditions, which suggested that gremlin may be involved in the pathogenesis of PDR.

In conclusion, the vitreous gremlin levels were elevated in the eyes with PDR compared with the eyes with idiopathic epimacular membrane. The expression of gremlin, fibronectin, and collagen IV was increased in RPE cells in the presence of high glucose, and the knockdown of gremlin significantly reduced the expression of fibronectin and collagen IV. The data suggest that gremlin may contribute to the pathological process of PDR. The present study extends our knowledge of the role of gremlin in the development PDR.

## Figures and Tables

**Table 1:** Clinical characteristics of the study population.

Clinical characteristics	PDR (*n* = 26)	Control (*n* = 22)	*p* value
Age (Y)			
Median (range)	55 (41-72)	66 (54-74)	*p* = 0.001
Gender			
Male	14 (54%)	12 (55%)	*p* = 0.401
Female	9 (41%)	13 (59%)	
Duration of diabetes (Y)			
Median (range)	9.6 (5-15)	—	
Fasting blood glucose (mmol/L)			
Median (range)	7.04 (4.13-8.98)	5.10 (4.16-6.0)	*p* < 0.001
Glycosylated hemoglobin (%)	6.8 (4.8-9.5)	—	
Received photocoagulation treatment	24 (92%)	—	
Received insulin treatment	23 (88.5%)	—	

*p* value was calculated by Mann–Whitney *U* test and chi-squared test between control and PDR cases.

## Data Availability

The data used to support the findings of this study are available from the corresponding author upon request.
